# Upward social comparison and social anxiety among Chinese college students: a chain-mediation model of relative deprivation and rumination

**DOI:** 10.3389/fpsyg.2024.1430539

**Published:** 2024-07-16

**Authors:** Lijuan Xu, Li Li

**Affiliations:** ^1^School of Education, Jiangxi Institute of Applied Science and Technology, Nanchang, China; ^2^Faculty of Psychology, Nanchang University, Nanchang, China

**Keywords:** upward social comparison, social anxiety, relative deprivation, rumination, Chinese college student

## Abstract

**Background:**

Social anxiety has consistently emerged as a prominent manifestation of mental health issues among college students. Though the relationship between upward social comparison and social anxiety has been extensively addressed in previous literature, little attention has been paid to the underlying mechanisms at play.

**Objective:**

The present study used a questionnaire survey to test whether upward social comparison may be associated with social anxiety in Chinese college students. The mediating role of relative deprivation and rumination was also examined.

**Methods:**

In total, 463 college students were recruited to complete four scales, including the Upward Social Comparison Scale, the Relative Deprivation Scale, the Ruminative Thinking Scale, and the Social Anxiety Scale.

**Results:**

The results show that upward social comparison was significantly positively correlated with relative deprivation, rumination, and social anxiety (*r* = 0.30, 0.31, and 0.27, respectively; *p* < 0.01). Second, relative deprivation was positively correlated with rumination and social anxiety (*r* = 0.40 and 0.17, respectively; *p* < 0.01). Finally, rumination was positively correlated with social anxiety (*r* = 0.47, *p* < 0.01). Moreover, upward social comparison positively predicts social anxiety, with an effect value of 0.12, while rumination plays a mediating role between upward social comparison and social anxiety, with an effect value of 0.07, and the 95% confidence interval for the indirect effect is 0.04–0.11. Separately, the chain mediation of relative deprivation and rumination had an effect value of 0.03, and the 95% confidence interval for the indirect effect is 0.02–0.05.

**Conclusion:**

This research highlights the relationship between upward social comparison and social anxiety in Chinese society and revealed the mediating mechanisms between them, deepening our understanding of how upward social comparison increases social anxiety.

## 1 Introduction

College students in China, as a group with special status given the current social development of the country, face changing interpersonal relationships in the family, school, and society and are going through the first social test of their lives, which usually brings about social anxiety. Social anxiety refers to an excessive panic and fear of being negatively evaluated by others when an individual is in a social situation, which results in them disconnecting themselves from various social activities, devaluing themselves, and potentially avoiding future social interactions (Rapee and Heimberg, 1997). Social anxiety can negatively affect an individual’s academic performance. Social anxiety is also closely related to various psychological problems, such as a low level of happiness (Guo and Huang, 2022; Xiao and Huang, 2022) and the presence of depression (Jiang et al., 2023), and it can cause behavioral problems like Internet addiction and aggression that directly affect an individual’s social adaptation (Gallagher et al., 2014; Rong and Yang, 2019; Jaiswal et al., 2020). Contemporary college students in China are a special group of youth with Chinese characteristics. Their vigorous growth will have an important impact on the future development of the country, and encouraging good social interactions is key to ensuring their life satisfaction and maintaining their physical and mental health (Xue et al., 2021). Research has indicated that college students are particularly vulnerable to experiencing social anxiety. Therefore, it is essential to thoroughly investigate the risk factors for social anxiety among college students and its internal mechanisms. Previous studies have suggested that upward social comparison (Li et al., 2007), relative deprivation (Xiong et al., 2022), rumination (Opwis et al., 2017), and self-concern (Leigh et al., 2021) may serve as influential factors in the development of social anxiety. Thus, based on prior research, this study developed a chain-mediation model to examine the mediating effects of relative deprivation and rumination on upward social comparison and social anxiety.

### 1.1 Upward social comparison and social anxiety

The concept of social comparison, first proposed by Festinger, refers to the fact that people unconsciously often compare themselves to others (Gilbert et al., 1995). Xing and Yu (2005) subdivided social comparisons into three different types according to the direction of the comparison: parallel comparisons, upward comparisons, and downward comparisons. Upward social comparison refers to the process by which individuals choose to compare themselves to others who are better than them in some way (Cao, 2022). Social comparison theory states that human beings are social animals, and there exists an outward-looking force within individuals that social psychologist Festinger called the inner drive. When individuals are in an uncertain environment, this inner drive will induce them to compare themselves to others in certain aspects and to a certain extent in order to increase their control over their external environment, leading to upward social comparisons (Buunk and Gibbons, 2007). Previous studies have shown that upward social comparison negatively impacts individual self-evaluation, such as by causing increased negative emotions, which is not conducive to individual mental health. Upward social comparison is an important factor affecting social anxiety (Tong et al., 2017; Zhang and Liu, 2022). College students frequently use their peers, seniors, or accomplished professionals as benchmarks during the process of personal development; however, they often experience a sense of inadequacy and pressure when engaging in upward social comparison, and they therefore strive to quickly bridge the perceived gap as result, leading to heightened social concerns (Li et al., 2007). Ma (2019) also arrived at a similar conclusion after conducting a survey of 350 college students in Heilongjiang. Therefore, the current study proposes the following:

*H1*: Upward social comparison has a significant positive predictive effect on college students’ social anxiety.

### 1.2 The mediating role of relative deprivation

Relative deprivation is defined collectively as the emotional experience and subjective perceptions generated by individuals when they perceive their inferiority in a certain aspect in comparison with other individuals around them. According to equity theory, the important variable that determines an employee’s job satisfaction is a sense of equity. When an individual finds that their rewards are less than those of other employees at the same level or who are performing similar work, they will feel unfairly treated (expressed by way of thoughts like “I have not received what I deserve”) and then subsequently relatively deprived, which can lead to negative emotions like depression and boredom and damage to their physical and mental health (Mark and Folger, 1984). As the emotional development of college students is relatively unstable, they have emerged as a group particularly susceptible to relative deprivation (Wang, 2011). College students with elevated levels of deprivation are more prone to manifest a negative emotional disposition (Li et al., 2019). Threat effect theory (Crawford, 2007) shows that, when an individual compares themselves to those who are better, they will place themselves in a disadvantageous position and thus have a strong sense of deprivation (Pan et al., 2023). Therefore, there is a close correlation between upward social comparison and relative deprivation. Previous studies have demonstrated that engaging in upward social comparison is a prerequisite for the emergence of relative deprivation, while individuals’ frequent engagement in upward social comparison positively predicts relative deprivation. Furthermore, according to classical relative deprivation theory, individuals typically assess their own situation by comparing themselves to their reference group; however, members of disadvantaged groups often find themselves at a disadvantage during this comparative process, leading to a sense of deprivation that hampers their psychological development (Mummendey et al., 1999). This sense of deprivation further contributes to various psychological adjustment issues like depression and social anxiety (Xiong and Ye, 2016). Specifically, feelings of relative deprivation encompass both cognitive and affective components (Smith et al., 2012). From a cognitive perspective, the core of relative deprivation is rooted in upward social comparison (Kim et al., 2018), which may lead individuals to perceive their own disadvantaged position and experience social anxiety during interactions. From an affective standpoint, individuals perceiving relative deprivation may undergo heightened negative emotions such as anger, resentment, and dissatisfaction, all of which have detrimental effects on individual mental well-being (Conrad et al., 2021). These studies provide robust support for predicting societal concerns regarding relative deprivation. Therefore, based on previous studies, this study considers relative deprivation to be a mediating variable and proposes the following:

*H2*: Relative deprivation plays a mediating role between upward social comparison and social anxiety among Chinese college students.

### 1.3 The mediating role of rumination

As a psychological concept, rumination entails the passive contemplation of causal factors following a negative experience as well as the contemplation of the event itself, the negative emotions stemming from it, and the potential adverse consequences, respectively. Overcoming these entrenched negative emotions poses challenges to an individual’s physical and mental well-being while also impeding effective problem-solving (Nolen-Hoeksema, 1991). Previous research has demonstrated a positive association between upward social comparison and rumination, suggesting that the former can serve as a direct predictor of the latter (Giordano et al., 2000; Robinson and Alloy, 2003; Antony et al., 2005). According to the stress-reactive rumination model (Robinson and Alloy, 2003), an individual’s level of rumination is likely to increase following their exposure to stressful events. When individuals engage in upward social comparison, they are more likely to experience an enhanced sense of inferiority and to generate negative self-evaluations. These feelings of inferiority and negative self-evaluations can serve as stressors that predict levels of rumination. The cognitive theory of social anxiety posits that negative cognitive biases play a pivotal role in the generation and perpetuation of social anxiety (Mobini et al., 2013). Empirical studies have also demonstrated that rumination serves as a robust predictor of social anxiety (Valena and Szentagotái-Tatar, 2015; Modini et al., 2018; Ma and An, 2019). As a cognitive processing mode involving the contemplation of conflicts (Schoofs et al., 2013), rumination engenders greater difficulties in adaptation, which are particularly observable in social interactions when confronted with obstacles, thereby leading to heightened levels of social anxiety (Fang and Sun, 2018). Consequently, we propose the following:

*H3*: Rumination acts as an intermediary mechanism through which upward social comparison influences social anxiety.

### 1.4 The chain-mediating role of relative deprivation and rumination

Further to the above, a significant association exists between relative deprivation and rumination. Empirical research has consistently demonstrated that the negative affective states and cognitive processes resulting from relative deprivation exert a profound influence on the process and outcomes of individual rumination (Gao et al., 2017; Pan et al., 2021)—that is, when individuals perceive themselves to be in an unfavorable position and experience emotions like anxiety and dissatisfaction, they tend to engage in repetitive recall of negative social events without employing rational analysis, thereby influencing both the process and outcomes of rumination. In addition, rumination also has a significant effect on relative deprivation. According to response style theory, an individual’s tendency to engage in rumination as a coping mechanism when faced with negative emotions (such as depression and anxiety) influences both the intensity and duration of these negative emotions (Li, 2019). By continuously contemplating and persistently focusing on disadvantageous events and negative emotions, rumination intensifies an individual’s perception of relative deprivation. Consequently, we hypothesize that upward social comparison affects social anxiety through the sequential mediation of relative deprivation and rumination.

*H4*: There is a chain-mediation effect between relative deprivation and rumination in the relationship between upward social comparison and social anxiety.

In summary, the objective of this study was to construct a chain-mediation model that elucidates the mechanisms of the influence of upward social comparison on social anxiety through relative deprivation and rumination. By doing so, we aim to provide a theoretical and empirical foundation for preventing and intervening in college students’ social anxiety.

## 2 Materials and methods

### 2.1 Participants

A questionnaire survey was conducted among undergraduate students from Jiangxi, Zhejiang, and Fujian provinces using a convenience sampling method and online questionnaires. Prior to the survey, participants were provided with a standardized guide that explained the purpose of the study and instructed them on how to answer the questions. A total of 463 valid questionnaires were finally collected after excluding invalid ones due to a short completion time and patterned responses. Among the respondents, 375 (80.99%) were female and 88 (19.01%) were male; additionally, 68 (14.69%) were freshmen, 178 (38.44%) were sophomores, 176 (38.01%) were juniors, and 41 (8.86%) were seniors, respectively. There were 34 (7.34%) students from single-parent families, but most students (429; 92.66%) came from non-single-parent families.

### 2.2 Measures

#### 2.2.1 Upward social comparison

The Upward Social Comparison Scale first developed by Gibbons and Buunk (1999) was subsequently revised by Chinese scholars (Bai et al., 2013) and tested among Chinese participants, where it was found to have good reliability and validity for measuring upward social comparison. The six-item questionnaire uses a 5-point scale to measure the level of upward social comparison. In this questionnaire, “1” means “strongly disagree” and “5” means “strongly agree.” The questionnaire does not incorporate reverse scoring. The higher the total score, the greater the level of upward social comparison by the participant. The overall consistency coefficient of the questionnaire was 0.88, indicating a high level of internal consistency and demonstrating strong reliability and validity, thus confirming its suitability for implementation within the scope of this research.

#### 2.2.2 Relative deprivation

The Relative Deprivation Scale used in this study was developed by Ma (2013). The scale contains four items (e.g., “I consistently perceive that others have acquired what rightfully belongs to me”) and uses a 6-point scale, with scores ranging from 1 point (“strongly disagree”) to 6 points (“strongly agree”). The higher the score, the greater the individual’s propensity for relative deprivation. In this study, Cronbach’s α coefficient for the Relative Deprivation Scale was 0.72, indicating strong validity.

#### 2.2.3 Rumination

The Ruminative Thinking Scale was originally developed by Nolen-Hoeksema and Morrow (1991) and subsequently adapted for use in China through localization and revision by Han and Yang (2009), who conducted a survey to assess its applicability within the Chinese context. The revised scale consists of 22 items with responses given on a 4-point scale ranging from 1 point (“rarely”) to 4 points (“frequently”) without any reverse scoring. The scale encompasses three dimensions: symptom meditation (items 1–4, 6, 8, 9, 14, 17–19, and 22), introspection (items 5, 10, 13, 15, and 16), and forced meditation (items 7, 11, 12, 20, and 21). The higher an individual scores on this scale, the more pronounced their tendency toward rumination. Cronbach’s α coefficient of the scale in this study was determined to be 0.96, indicating a high level of reliability and validity.

#### 2.2.4 Social anxiety

The Chinese version of the Social Anxiety Scale, based on the Interaction Anxiousness Scale developed by Leary (1983), was used to measure the social anxiety of participants (Leary, 1983; Peng et al., 2004). The scale contains 15 single-dimensional items (e.g., “I get nervous when I must talk to a teacher or boss”), and participants indicate their agreement on a 5-point scale (1 = “little/no agreement”; 5 = “high/complete agreement”). After items 3, 6, 10, and 15 are reverse-scored, participants’ social anxiety is assessed by summing the scores for each item, with higher scores indicating greater social anxiety. In this study, Cronbach’s α coefficient of the scale was determined to be 0.86.

### 2.3 Procedure

The research design used in this study was a questionnaire survey. Before participation, participants were informed that their participation was completely voluntary and received information about the study. We placed emphasis on them answering all questions honestly and confidentially, and their anonymity was ensured. Under the guidance of trained investigators, participants completed the Upward Social Comparison Scale, the Relative Deprivation Scale, the Ruminative Thinking Scale, and the Social Anxiety Scale. We obtained a final representative sample of 463 valid questionnaires.

## 3 Results

### 3.1 Common method variance analysis

Before testing the hypotheses, we analyzed the common method variance by performing Harman’s one-factor test. According to previous studies, if a common factor accounts for more than 40% of the total variance, it indicates that there is a common method variance. In this study, the results showed that the eigenvalue of seven factors exceeded 1, with the first factor accounting for 32.62% of the total variance. Additionally, the results obtained from confirmatory factor analysis indicated that the fit indices were not satisfactory, with χ^2^/df = 3.03, RMSEA = 0.07, CFI = 0.83, GFI = 0.75, NFI = 0.78, and TLI = 0.83. These findings indicate that the problem of common method variance in this study was not serious.

### 3.2 Correlation analysis of upward social comparison, relative deprivation, rumination, and social anxiety

Descriptive statistics and Pearson’s correlations for all of the assessed variables are presented in Table 1. This table shows that upward social comparison was significantly and positively correlated with social anxiety (*r* = 0.27, *p* < 0.001). Similarly, relative deprivation was significantly and positively associated with upward social comparison and social anxiety (*r*_1_ = 0.30, *p* < 0.001; *r*_2_ = 0.17, *p* < 0.001), while rumination was found to be significantly correlated with both upward social comparison and social anxiety (*r*_1_ = 0.31, *p* < 0.001; *r*_2_ = 0.47, *p* < 0.001). Finally, relative deprivation demonstrated a substantial positive relationship with rumination (*r* = 0.40, *p* < 0.001).

**Table 1:** Descriptive statistics and Pearson correlations between the study variables.

Variable	*M* ± SD	1	2	3	4	5
1.Sex	1.81 ± 0.39	–				
2.Upward social comparison	3.09 ± 0.80	−0.23	–			
3.Relative deprivation	2.58 ± 0.86	−0.17^**^	0.30^***^	–		
4.Rumination	1.95 ± 0.51	−0.21	0.31^***^	0.40^***^	–	
5.Social anxiety	3.15 ± 0.61	0.12^**^	0.27^***^	0.17^***^	0.47^***^	–

^*^p < 0.05, ^**^p < 0.01, ^***^p < 0.001. Sex: 1 = male, 2 = female.

### 3.3 Analysis of mediation effects

**Figure 1:**
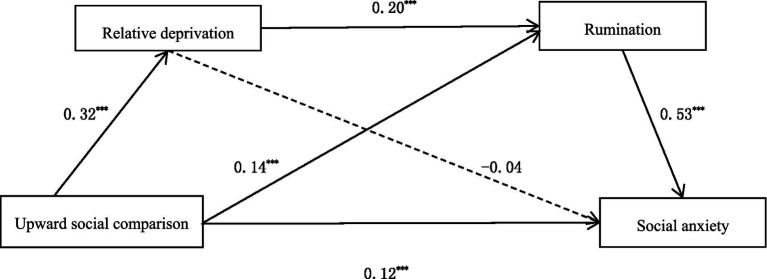
Serial mediation model (*p < 0.05, **p < 0.01, ***p < 0.001).

A serial mediation model was tested for two indirect effects, hypothesized as follows: first, upward social comparison increases social anxiety via rumination; second, upward social comparison influences social anxiety via relative deprivation and rumination (Figure 1).

As shown in Table 2, after controlling for the effect of sex, the results presented positive effects of upward social comparison on relative deprivation (β = 0.32, *t* = 6.67, *p* < 0.001) and rumination (β = 0.14, *t* = 4.86, *p* < 0.001). There was also a positive relationship between relative deprivation and rumination (β = 0.20, *t* = 7.67, *p* < 0.001). Furthermore, both upward social comparison and rumination demonstrated a positive relationship with social anxiety among the participants (β_1_ = 0.12, *t*_1_ = 3.48, *p* < 0.001; β_2_ = 0.53, *t*_2_ = 9.69, *p* < 0.001). As a result, we determined that the chain-mediated effect of upward social comparison through relative deprivation and rumination on social anxiety is significant.

**Table 2:** Regression analysis of the research variables.

Regression equation		Overall fit index		Significance of regression coefficients		
Outcome	Predictor	*R* ^2^	*F*	*β*	SE	*t*
Relative deprivation	Upward social comparison	0.09	44.48^***^	0.32	0.05	6.67^***^
Rumination	Upward social comparison	0.20	57.36^***^	0.14	0.03	4.86^***^
	Relative deprivation			0.20	0.03	7.67^***^
Social anxiety	Upward social comparison	0.24	47.95^***^	0.12	0.03	3.48^***^
	Relative deprivation			−0.04	0.32	−1.19
	Rumination			0.53	0.05	9.69^***^

^*^p < 0.05, ^**^p < 0.01, ^***^p < 0.001.

The PROCESS macro (Model 6) by Hayes (2013) was applied to examine the possible effects of relative deprivation and rumination as mediators between upward social comparison and social anxiety. This macro tests the mediation effect by using the bootstrap method to create a 95% confidence interval (CI) (5,000 bootstrapped resamples were used in this study). If the interval does not contain a 0, the mediation effect is considered to be reliable (Preacher and Hayes, 2008). As shown in Table 3, the direct effect of upward social comparison on social anxiety was significant (*f* = 0.12; 95% CI, 0.05–0.18), confirming H1. Additionally, Table 3 shows that the single-mediation role of rumination and the chain-mediating role of relative deprivation and rumination, respectively, were small but significant (*f* = 0.07; 95% CI, 0.04–0.11) (*f* = 0.03; 95% CI, 0.02–0.05), verifying both H3 and H4. Meanwhile, the indirect effect of upward social comparison on social anxiety through relative deprivation was not significant (*f* = −0.01, 95% CI, [−0.04, 0.01]).

**Table 3:** Direct and indirect effects of upward social comparison on social anxiety.

Model pathways	Effect size	Boot SE	95% LLCI	95% ULCI	Ratio
**Direct effect**					
Upward social comparison → social anxiety	0.12	0.03	0.05	0.18	57.14%
**Indirect effect**					
Path 1: upward social comparison → relative deprivation → social anxiety	−0.01	0.01	−0.04	0.01	−4.76%
Path 2: upward social comparison → rumination → social anxiety	0.07	0.02	0.04	0.11	33.33%
Path 3: upward social comparison → relative deprivation → rumination → social anxiety	0.03	0.01	0.02	0.05	14.29%
	0.21	0.03	0.14	0.28	

LLCI, lower limit of the confidence interval; SE, standard error; ULCI, upper limit of the confidence interval.

## 4 Discussion

This study examined in comprehensive fashion the associations between upward social comparison, relative deprivation, rumination, and social anxiety among college students in China. The findings demonstrate that upward social comparison significantly predicts social anxiety in Chinese college students through the mediating effects of relative deprivation and rumination. These results not only contribute to the existing literature on social anxiety among college students but also offer valuable insights for preventing and alleviating this condition.

### 4.1 The direct effect of upward social comparison on social anxiety

The findings show that upward social comparison positively predicts college students’ levels of social anxiety, indicating that greater degrees of upward social comparison are associated with an increased likelihood of experiencing elevated levels of social anxiety. With this finding, H1 was verified, consistent with results of previous research (Li et al., 2007; Liu, 2021). Social comparison is a prevalent psychological phenomenon whereby individuals frequently enhance their self-awareness and engage in self-evaluation (Gilbert et al., 1995). Compared to downward social comparison, upward social comparison is more likely to have a detrimental impact on the psychological stability of college students and lead to unfavorable outcomes, such as cognitive dissonance (Tong et al., 2017). In the process of development, college students typically adopt their classmates or distinguished individuals in their field as role models; however, they often experience heightened social anxiety through upward social comparison and the pressure to quickly bridge the perceived gap with others. The social comparison theory has been substantiated by empirical research, with upward social comparison found to positively predict the level of anxiety experienced by individuals during real-life social interactions (Wen et al., 2019). The contrast effect suggests that individuals will experience negative emotions, such as feeling threatened, distressed, or frustrated, when they perceive information that is inconsistent with the comparison object (Han and Chi, 2012). Therefore, under the influence of the contrast effect, the greater the degree of upward social comparison, the higher the level of anxiety individuals will experience. It is evident that upward social comparison plays a significant role in the development of social anxiety experienced by college students. Therefore, educational institutions and families should foster a healthy social comparison perspective among college students, imparting the ability to approach diverse social situations with composure and objectivity. Furthermore, they should equip students with effective strategies for alleviating social anxiety.

### 4.2 The indirect effects of upward social comparison on social anxiety

#### 4.2.1 The mediating role of relative deprivation between upward social comparison and social anxiety

In this study, the mediating role of relative deprivation in the relationship between upward social comparison and social anxiety among college students did not yield a significant effect, so H2 remains unconfirmed. While upward social comparison positively predicts social anxiety in college students, its predictive effect on social anxiety through relative deprivation is not significant. Previous research has demonstrated that, when an individual’s affordability significantly surpasses relative deprivation, it generally stimulates reasonable competition among individuals (Wang, 1988). During such circumstances, a rational disparity can effectively mobilize an individual’s initiative. Conversely, when relative deprivation surpasses or even exceeds an individual’s capacity, it tends to evoke hostile emotions, self-negation, and antagonism toward society. Data analysis revealed that the level of relative deprivation among college students in this study was 2.58 ± 0.86, suggesting a moderate degree of relative deprivation and an inherent capacity to cope with challenges, thereby fostering healthy competition and minimizing negative emotional outcomes.

#### 4.2.2 The mediating role of rumination between upward social comparison and social anxiety

Our findings show that rumination serves as a significant mediating factor between upward social comparison and social anxiety among college students; in other words, upward social comparison has been found to be a significant predictor of social anxiety among college students through the mediating effect of rumination, confirming H3. The frequency of engaging in upward social comparison correlates positively with the perceived stress level, indicating a higher level of stress experienced by individuals. The stress-response model of rumination suggests that the greater the perceived stress of an individual, the higher their level of rumination (Robinson and Alloy, 2003). Furthermore, rumination exhibited a positive predictive effect on social anxiety in this study, aligning with the findings of Ni et al. (2021) and Kocovski et al. (2005). Rumination directs an individual’s attention toward negative events, thereby exacerbating or prolonging negative emotional reactions during interpersonal communication. This propensity to engage in a negative thinking cycle increases the likelihood of making pessimistic predictions about future interpersonal interactions and subsequently intensifies levels of social anxiety. Moreover, according to the cognitive theory of social anxiety, negative cognitive biases play a pivotal role in both the development and perpetuation of social anxiety disorder. Rumination, as a cognitive mode of processing conflicts (Schoofs et al., 2013), is inclined to elicit elevated levels of social anxiety in individuals when they are confronted with social obstacles (Fang and Sun, 2018). Therefore, for college students exhibiting high levels of rumination, educational institutions should implement positive rumination training programs to facilitate cognitive restructuring, foster constructive interpretations, and promote self-affirmation and positive evaluations of oneself and others. Parents can foster positive thinking in their children through engaging in constructive conversations. The implementation of these measures appears to have yielded both improvements in positive perceptions and reductions in rumination levels.

#### 4.2.3 The chain-mediating effect of relative deprivation and rumination between upward social comparison and social anxiety

This study provides further evidence that upward social comparison is associated with college students’ social anxiety through a chain-mediating process involving relative deprivation and rumination. Specifically, the results suggest that upward social comparison first triggers feelings of relative deprivation, which in turn leads to increased rumination. Finally, this heightened level of rumination contributes to greater levels of social anxiety among college students, thus supporting H4. Research has indicated that college students have emerged as a vulnerable demographic in terms of experiencing relative deprivation (Wang, 2011). According to threat effect theory (Schoofs et al., 2013), when college students engage in upward social comparisons, they tend to position themselves at a relative disadvantage, thereby eliciting a sense of deprivation. The negative affective and cognitive experiences arising from relative deprivation exert a significant effect on the process and outcomes of individual rumination (Gao et al., 2017; Pan et al., 2021). Putting it differently, when individuals perceive themselves as being disadvantaged or treated unfairly, they are more likely to exhibit cognitive biases (Mummendey et al., 1999), leading to a diminished capacity for rational analysis and an increased propensity to repetitively recall negative social events. This can result in rumination. Rumination, as an ineffective cognitive emotion-regulation strategy, may be a stronger predictor of changes in social anxiety (Brozovich et al., 2015; Opwis et al., 2017). Collectively, these findings suggest the necessity of acknowledging the significant role of relative deprivation and rumination in the association between upward social comparison and social anxiety among college students. Therefore, educators should provide guidance designed to foster college students’ proper values, promptly monitor student dynamics, endeavor to mitigate the negative psychological issues arising from relative deprivation resulting from upward social comparison, alleviate levels of social anxiety among college students, and enhance college students’ mental well-being. Simultaneously, it is imperative for college students to adopt a rational and accurate perspective when evaluating the outcomes of upward social comparison, to cultivate an appropriate understanding of social comparison, and to approach challenges with a positive mindset. Finally, it is imperative for society to enhance the notion of equity and establish a robust mechanism for coordinating fair and equitable interests.

### 4.3 Research implications and limitations

The present study focused on investigating the social anxiety experienced by college students and delved into examining the relationship between upward social comparison and social anxiety among college students, along with its internal mechanisms. Our study’s findings indicate that upward social comparison can be a significant predictor of college students’ social anxiety, with rumination playing a mediating role in this process, while relative deprivation and rumination jointly play a chain-mediating role. This research holds substantial practical implications for addressing the issue of social anxiety, which significantly affects the socioemotional development of college students. First, the results contribute to the cognitive–behavioral framework theory of social anxiety and facilitate comprehension of the association between upward social comparison and social anxiety within the context of relative deprivation and individual rumination. Second, they offer practical insights for addressing upward social comparison and intervening in social anxiety among college students. Educators should closely monitor disadvantaged students and those with high levels of rumination, taking appropriate steps to alleviate their relative deprivation and rumination, such as helping them to recognize their strengths through praise and encouragement and providing active rumination training to indirectly reduce social anxiety (Yang, 2019).

However, this study does possess certain limitations. First, in terms of research design, a cross-sectional approach was employed, which precludes the exploration of any causal relationships among the variables. In future research, longitudinal methods could be employed to investigate further the association between upward social comparison and social anxiety as well as the underlying mechanisms. Additionally, this study focused solely on the mediating role of relative deprivation and rumination in the association between upward social comparison and social anxiety, thus omitting any investigation of the differential impacts of other variables in this relationship. In future research, it is recommended that multiple variables be incorporated alongside upward social comparison and social anxiety to explore their interplay, thereby uncovering additional mediators or moderators that may contribute to reducing the levels of social anxiety.

## 5 Conclusion

To conclude, this study has shown that upward social comparison exerts both direct and indirect positive effects on college students’ social anxiety. Rumination serves as a partial mediator, while relative deprivation and rumination act as sequential mediators, respectively, in the relationship between upward social comparison and the experience of social anxiety.

## Data availability statement

The original contributions presented in the study are included in the article/Supplementary material, further inquiries can be directed to the corresponding author.

## Ethics statement

The studies involving humans were approved by Nanchang University – BioMedicineEthics Committee Approval. The studies were conducted in accordance with the local legislation and institutional requirements. The participants provided their written informed consent to participate in this study.

## Author contributions

LX: Writing - original draft, Conceptualization. LL: Writing - review & editing, Supervision.
