# Supplementary material for: Upward social comparison and social anxiety among Chinese college students: a chain-mediation model of relative deprivation and rumination
Source: Front Psychol. 2024 Jul 16;15:1430539. doi: 10.3389/fpsyg.2024.1430539 (PMC11286571; doi:10.3389/fpsyg.2024.1430539)
Supplement: Supplementary file 1 [file Data_Sheet_1.docx]

Supplementary Material

Upward Social Comparison and Social Anxiety Among Chinese College Students: A Chain-mediation Model of Relative Deprivation and Rumination

Lijuan Xu^1^, Li Li^2*^

*** Correspondence:** Li Li: [lancylili@ncu.edu.cn](mailto:lancylili@ncu.edu.cn)

# Introduction

Dear classmates, please help me with my academic research by completing this questionnaire. Your answers will provide me with valuable research data. This questionnaire is anonymous, and there is no right or wrong answer—instead, please choose the ones that best suit you. Thank you for participating in this survey!

The questionnaire consists of four parts composed of four, six, 15, and 22 questions, respectively. The questionnaire should take 3 min to complete. Thank you again for your attention.

# Basic Information

1. Your sex : ① Male ② Female

2. Your grade: ① Freshman ② Sophomore ③ Junior ④ Senior

3. Single-parent family or not : ① From a single-parent family ② Not from a single-parent family

# Relative Deprivation Scale ( D )

Please read the following items carefully and choose the appropriate level of agreement with each statement.

**d1** 4. Given all the efforts I've made, my life should be better than it is now.

① Strongly disagree ② Disagree ③ Somewhat disagree ④ Somewhat agree ⑤ Agree ⑥ Strongly agree

**d2** 5. I always feel like someone else is taking what's rightfully mine.

① Strongly disagree ② Disagree ③ Somewhat disagree ④ Somewhat agree ⑤ Agree ⑥ Strongly agree

**d3** 6. Compared to the people around me, I am underperforming in every aspect of my life.

① Strongly disagree ② Disagree ③ Somewhat disagree ④ Somewhat agree ⑤ Agree ⑥ Strongly agree

**d4** 7. Most of the rich people in society have made their fortune through disgraceful means.

① Strongly disagree ② Disagree ③ Somewhat disagree ④ Somewhat agree ⑤ Agree ⑥ Strongly agree

# Upward Social Comparison Scale ( E )

Have you ever compared yourself to others? Please choose the appropriate level of agreement with each of the following statements.

**e1** 8. In daily life, I often like to compare myself with those who do better than me.

1. Strongly disagree ② Somewhat disagree ③ Uncertain ④ Somewhat agree ⑤ Strongly agree

**e2** 9. When I evaluate my current situation in life or study, I often compare myself with those who are doing better than me.

1. Strongly disagree ② Somewhat disagree ③ Uncertain ④ Somewhat agree ⑤ Strongly agree

**e3** 10. When things go badly, I often think of those who are doing better than me.

1. Strongly disagree ② Somewhat disagree ③ Uncertain ④ Somewhat agree ⑤ Strongly agree

**e4** 11. When I evaluate my own communication skills, I prefer to compare myself to those who have better communication skills than I do.

1. Strongly disagree ② Somewhat disagree ③ Uncertain ④ Somewhat agree ⑤ Strongly agree

**e5** 12. When I think about whether I can do anything well, I often compare myself to those who are better than me.

1. Strongly disagree ② Somewhat disagree ③ Uncertain ④ Somewhat agree ⑤ Strongly agree

**e6** 13. There are times in my life when I compare myself to those who are more successful than me.

1. Strongly disagree ② Somewhat disagree ③ Uncertain ④ Somewhat agree ⑤ Strongly agree

# Social Anxiety Scale ( F )

How do you behave in social interactions? Please choose the appropriate level of agreement with each of the following statements.

**f1** 14. I feel nervous even at informal gatherings.

① Little/no agreement ② Some agreement ③ Moderate agreement ④ Significant agreement ⑤ High/complete agreement

**f2**  15. I don't feel at ease with a group of strangers.

① Little/no agreement ② Some agreement ③ Moderate agreement ④ Significant agreement ⑤ High/complete agreement

**f3** 16. I usually feel relaxed when talking to a person of the opposite sex.

① Little/no agreement ② Some agreement ③ Moderate agreement ④ Significant agreement ⑤ High/complete agreement

**f4** 17. I feel nervous when I must talk to my teacher or my boss.

① Little/no agreement ② Some agreement ③ Moderate agreement ④ Significant agreement ⑤ High/complete agreement

**f5** 18. Parties often make me feel anxious and out of place.

① Little/no agreement ② Some agreement ③ Moderate agreement ④ Significant agreement ⑤ High/complete agreement

**f6** 19. I am less socially shy than most people.

① Little/no agreement ② Some agreement ③ Moderate agreement ④ Significant agreement ⑤ High/complete agreement

**f7**  20. I often feel nervous when talking to people of the same sex I don't know very well.

① Little/no agreement ② Some agreement ③ Moderate agreement ④ Significant agreement ⑤ High/complete agreement

**f8** 21. I get nervous during job interviews.

① Little/no agreement ② Some agreement ③ Moderate agreement ④ Significant agreement ⑤ High/complete agreement

**f9** 22. I wish I had more confidence in socializing.

① Little/no agreement ② Some agreement ③ Moderate agreement ④ Significant agreement ⑤ High/complete agreement

**f10** 23. I rarely feel anxious in social situations.

① Little/no agreement ② Some agreement ③ Moderate agreement ④ Significant agreement ⑤ High/complete agreement

**f11** 24. Generally speaking, I am a shy person.

① Little/no agreement ② Some agreement ③ Moderate agreement ④ Significant agreement ⑤ High/complete agreement

**f12** 25. I feel nervous when talking to an attractive person of the opposite sex.

① Little/no agreement ② Some agreement ③ Moderate agreement ④ Significant agreement ⑤ High/complete agreement

**f13** 26. I feel nervous when I call someone I don't know very well.

① Little/no agreement ② Some agreement ③ Moderate agreement ④ Significant agreement ⑤ High/complete agreement

**f14**  27. I feel nervous when talking to authority figures.

① Little/no agreement ② Some agreement ③ Moderate agreement ④ Significant agreement ⑤ High/complete agreement

**f15** 28. Even when I am in a group of people quite different from me, I usually feel at ease.

① Little/no agreement ② Some agreement ③ Moderate agreement ④ Significant agreement ⑤ High/complete agreement

# Ruminative Thinking Scale **( G )**

Do you wrestle with certain thoughts repeatedly? Please choose the appropriate level of agreement with each of the following statements.

**a1** 29. I often think about how lonely I am.

① Rarely ② Sometimes ③ Often ④ Frequently

**a2** 30. I often think, "if I can't stop thinking about this, then I can't keep doing what I'm doing."

① Rarely ② Sometimes ③ Often ④ Frequently

**a3** 31. I often think about my feelings of fatigue and pain.

① Rarely ② Sometimes ③ Often ④ Frequently

**a4** 32. I often think, "how hard it is to concentrate."

① Rarely ② Sometimes ③ Often ④ Frequently

**b1** 33. I often wonder what I could have done to cause this.

① Rarely ② Sometimes ③ Often ④ Frequently

**a5** 34. I often think about how passive and unmotivated I am.

① Rarely ② Sometimes ③ Often ④ Frequently

**c1** 35. I often analyze recent events to understand why I feel depressed.

① Rarely ② Sometimes ③ Often ④ Frequently

**a6** 36. I often think I'm numb to other things.

① Rarely ② Sometimes ③ Often ④ Frequently

**a7** 37. I often wonder why I'm so frustrated.

① Rarely ② Sometimes ③ Often ④ Frequently

**b2** 38. I often wonder why I always do this.

① Rarely ② Sometimes ③ Often ④ Frequently

**c2** 39. I often wonder alone why this is so.

① Rarely ② Sometimes ③ Often ④ Frequently

**c3** 40. I often write down what I'm thinking and analyze it.

① Rarely ② Sometimes ③ Often ④ Frequently

**b3** 41. I often think about the present situation and wish it would get better.

① Rarely ② Sometimes ③ Often ④ Frequently

**a8** 42. I often think that, if this feeling persists, I won't be able to concentrate.

① Rarely ② Sometimes ③ Often ④ Frequently

**b4** 43. I often wonder why I have these problems but others don't.

① Rarely ② Sometimes ③ Often ④ Frequently

**b5** 44. I often wonder why I can't handle things better.

① Rarely ② Sometimes ③ Often ④ Frequently

**a9** 45. I often wonder why I feel so sad.

① Rarely ② Sometimes ③ Often ④ Frequently

**a10** 46. I often think about my shortcomings, failures, fault and mistakes.

① Rarely ② Sometimes ③ Often ④ Frequently

**a11** 47. I often think, "I can't get up to anything."

① Rarely ② Sometimes ③ Often ④ Frequently

**c4** 48. I often analyze my own personality to understand why I feel depressed.

① Rarely ② Sometimes ③ Often ④ Frequently

**c5**  49. I often go somewhere alone to think about my feelings.

① Rarely ② Sometimes ③ Often ④ Frequently

**a12**  50. I often think about how angry I am with myself.

① Rarely ② Sometimes ③ Often ④ Frequently

Note:

1. Sex: 1 = male, 2 = female. 2. grade: 1 = freshmen, 2 = sophomore, 3 = junior, 4 = senior. 3. from single parent families or not: 1 =from single parent families , 2 = not from single parent families.

4. D = Relative Deprivation Scale, E = Upward Social Comparison Scale, F = Social Anxiety Scale, G = Ruminative Thinking Scale, a (total) = a1+a2+a3+a4+a5+a6+a7+a8+a9+a10+a11+a12, b (total) = b1+b2+b3+b4+b5, c (total) = c1+c2+c3+c4+c5.
